# Effects of dietary supplementation with omega-3 fatty acid-rich linseed on the reproductive performance of ewes in subtropical climates

**DOI:** 10.3389/fvets.2024.1398961

**Published:** 2024-06-24

**Authors:** P. Akhtar, J. S. Rajoriya, A. K. Singh, B. K. Ojha, A. K. Jha, A. Bisen, Nitin K. Bajaj, M. K. Ahirwar, A. Raje, A. P. Singh, S. S. Peepar, A. K. Mishra, Rahul Katiyar, Jayanta Chamuah, Mahak Singh

**Affiliations:** ^1^Shri Sadguru Gau Seva Kendra, Jankikund, Sadguru Netra Chikitsalaya, Chitrakoot, MP, India; ^2^Department of Veterinary Gynaecology and Obstetrics, NDVSU-College of Veterinary Science and Animal Husbandry, Rewa, MP, India; ^3^Department of Veterinary Physiology and Biochemistry, NDVSU-College of Veterinary Science and Animal Husbandry, Rewa, MP, India; ^4^Department of Animal Nutrition, NDVSU-College of Veterinary Science and Animal Husbandry, Rewa, MP, India; ^5^Department of Animal Genetics and Breeding, NDVSU-College of Veterinary Science and Animal Husbandry, Rewa, MP, India; ^6^Department of Veterinary Physiology and Biochemistry, NDVSU-College of Veterinary Science and Animal Husbandry, Mhow, MP, India; ^7^Department of Veterinary Pharmacology and Toxicology, NDVSU-College of Veterinary Science and Animal Husbandry, Rewa, MP, India; ^8^Division of Animal Reproduction, ICAR-Indian Veterinary Research Institute, Bareilly, UP, India; ^9^Department of Livestock Production Management, NDVSU-College of Veterinary Science and Animal Husbandry, Rewa, MP, India; ^10^ICAR Research Complex for NEH Region, Umiam, Meghalaya, India; ^11^ICAR-NRC-Mithun, Medziphema, Nagaland, India; ^12^ICAR Nagaland Centre, Medziphema, Nagaland, India

**Keywords:** linseed, ω-3 PUFAs, dietary supplementation, reproductive performance, ewes, subtropical

## Abstract

The present study evaluated the effects of omega-3 (ω-3) fatty acid-rich linseed supplementation on the reproductive performance, endocrine profile, and biochemical profile of ewes reared in subtropical climates. Forty-eight acyclic and clinically healthy Marwari sheep, aged 1.5–2.5 years with no parity, were divided into four groups (*n* = *n* = 12 in each). Ewes in the control group (group I) were fed only a basal feed, whereas ewes in the treatment groups II, III, and IV were fed the basal diet along with 10%, 15%, and 20% linseed, respectively, daily on a dry matter basis. The experiment was conducted during the typical breeding season (October–November) of the sheep. The estrus induction rate was significantly higher (*p* < 0.05) in all treatment groups than in the control group. The estrus induction interval was significantly lower (*p* < 0.05) in group III. The conception rate in group I was significantly lower (*p* < 0.05). In addition, ewes in the control group had a significantly lower (*p* < 0.05) lambing rate than all treatment groups. Serum progesterone concentrations differed significantly (*p* < 0.05) between the control and the treatment groups on days 15, 30, 45, and 60 of supplementation. On treatment days 15 and 30, the serum estrogen concentrations were significantly higher (*p* < 0.05) in all treatment groups compared to that in group I. In all treatment groups, monounsaturated fatty acid (MUFA) decreased significantly (*p* < 0.05), whereas polyunsaturated fatty acid (PUFA) increased significantly (*p* < 0.05) from day 15 onward. In conclusion, by providing 15% dietary linseed supplementation to ewes, their reproductive performance can be improved in subtropical climates. Future studies are recommended to further elucidate the role of linseed supplementation in sheep reproduction in subtropical climates.

## 1 Introduction

Sheep play an important role in the livelihood and nutritional security of small and marginal farmers globally. The reproductive efficiency of sheep is compromised in subtropical climates because of nutritional and environmental factors. Improving the reproductive performance of sheep is important to improve and utilize limited animal resources and meet humans' need for quality animal protein. It has been previously reported that flushing ewes before breeding can improve folliculogenesis, ovulation, and lambing rates in sheep (1, 2). Nutritional interventions are preferred over hormonal interventions to augment the reproductive efficiency in livestock. It is generally accepted that nutrition not only increases the strength of animals but also determines their reproductive efficiency depending on the fatty acid (FA) composition of the diet (3, 4). Liel et al. (5) reported that FA calcium soap supplementation increases the number and size of ovarian follicles and serum progesterone concentrations in cyclic sheep. The addition of fatty foods to the diet has long been used to reduce postpartum depression, improve energy balance to increase milk production, and significantly improve fertility in lactating cows (6–8). Therefore, attention has been focused on determining the reproductive effects of various FAs, particularly long-chain polyunsaturated fatty acids (PUFAs), such as omega-3 (ω-3) and omega-6 (ω-6) FAs (8, 9).

Previous studies have shown that dietary supplementation of ω-3 PUFAs may have beneficial effects on the developmental processes of humans and animals (10). Mattos et al. (3) showed that ω-3 PUFAs can reduce uterine prostaglandin F2α (PGF2α) release and reduce the sensitivity of the corpora lutea (CL) to PGF2α, potentially aiding in preventing luteolysis and fostering the establishment of cow pregnancy. Omega-3 PUFA supplementation is associated with an increased conception rate, follicular turnover and growth, ovulation, CL size, and steroidogenesis (11, 12). Moreover, increasing the long-chain ω-3 PUFA intake in the later stages of pregnancy prolongs the pregnancy and prevents behavioral abnormalities, such as the time taken for newborns to stand and breastfeed (13, 14). Other studies have also highlighted the role of nutrition in improving the performance of farm animals such as buffalos (15–17), horses (18), and pigs (19, 20). Omega-3 PUFAs used for animals to enhance their fertility can be obtained from varied sources. Flaxseed or linseed is one of the commonly available ingredients that are rich in ω-3 FAs and short-chain PUFAs (21). Although flaxseed supplementation in cows, buffalos, and pigs has been extensively researched, information on its effects on nulliparous sheep is limited. In addition, studies on the effect of flaxseed supplementation on sheep reproduction in subtropical climates are lacking (2). Therefore, this study aimed to examine the effects of flaxseed supplementation on the fertility, endocrine profile, hematological profile, and serum FA profile of sheep in a subtropical climate.

## 2 Materials and methods

### 2.1 Experimental location

The experiment was conducted at the Department of Veterinary Obstetrics and Gynecology, College of Veterinary Science and Animal Husbandry, Rewa, Madhya Pradesh, India. Madhya Pradesh is located in central India, between latitudes 170° and 25° North and longitudes 72° and 85° East. It has a warm subtropical climate with an average annual precipitation of 1,128 mm. The temperature ranges from 4°C in winter to 45°C in summer.

### 2.2 Selection of animal

In this experiment, 48 healthy Marwari sheep, aged 1.5–2.5 years, were used. The average body weight of the animals was 42.38 ± 1.25 kg, and the body condition score was 3.6 ± 0.07 on a scale of 1–5. The animals were kept under a semi-intensive system, which included grazing for 6–8 h per day. Before starting the experiment, the animals were confirmed to be acyclic and healthy based on the following conditions: (1) no penetration behavior of the ram for 21 consecutive days; (2) absence of any signs of estrus (e.g., mucus discharge or frequent urination); (3) absence of the ovarian CL on ultrasound examination (7.5 MHz, Mindray, USA) twice at an interval of 10 days, and (4) serum progesterone spectrum (P_4_) of < 1 ng/mL based on the blood samples taken 10 days before the beginning of the experiment. The experiment was performed during the ewes' breeding season (October to November). The study was approved by the Institutional Animal Ethics Committee of the College of Veterinary Science, Rewa, Madhya Pradesh, India.

### 2.3 Experimental design and feeding regime

Forty-eight sheep were divided into four equal groups. The control group (group I) was fed only a basal diet. Sheep in the treatment groups, namely groups II, III, and IV, were given concentrated feeds containing 10%, 15%, and 20% flaxseed, respectively. All groups were fed isonitrogenous and isocaloric feed (Table 1). Before starting the actual diet, 7 days were provided for adaptation to the new diet, during which the amount of linseed was gradually increased in the basal diet. The day of initiation of the acclimatization period and the day of commencement of the actual feeding are denoted as day −7 and day 0, respectively. The ewes in the treatment groups were administered the flaxseed-supplemented diet for 60 days.

**Table 1:** Composition (%) of the ration fed to the experimental animals.

**Ingredients**	**Group I**	**Group II**	**Group III**	**Group IV**
Whole linseed	0	10	15	20
Soybean meal	10	10	10	10
Wheat bran	30	30	30	30
Rice police	28	28	28	28
Mustard cake	30	20	15	10
Mineral mixture	1.5	1.5	1.5	1.5
Common salt	0.5	0.5	0.5	0.5
Total	100	100	100	100
CP (%)	23.4	22.94	22.85	22.84
TDN (%)	69.6	70.1	70.35	70.6

CP, crude protein; TDN, total digestoble nutrient.

### 2.4 Estrus detection and breeding

The onset of estrus in ewes was visually detected by observing their interaction with vasectomized rams (those that have normal sex libido but cannot impregnate), which were walked for 30 min two times a day. Ewes that were in heat were bred twice, at an interval of 12 h, with proven rams. Six rams were used in the rotation for natural breeding. If an ewe remained motionless when mounted on by a ram, it was considered to be in heat. The number of animals entering estrus (estrus induction rate) in each group was recorded. The estrus induction interval is defined as the time between the start of treatment and the first removal of the ram. The conception rate is calculated as the ratio of the number of conceived (or pregnant) ewes in each group to the total number of ewes. The lambing rate is calculated as the ratio of the number of ewes that lambed in each group to the total number of mated ewes. Pregnancy in mated ewes was tested by lutein assay (ELISA) on day 18 of mating and confirmed by transabdominal ultrasonography (7.5 MHz, Mindray, USA) on day 28 of mating. During the lambing period, the sheep were monitored 24/7, and each calving time was recorded. The gestation length was calculated from the day of first breeding to the day of lambing.

### 2.5 Blood collection

During the feeding trial, blood samples were collected at −7, 0, 15, 30, 45, and 60 days of the experiment. The blood was collected between 7:00 am and 08:00 am via jugular venipuncture and was stored in vacutainers containing clot-activating factors to obtain serum. On day 18 post-estrus, blood samples were collected from the animals (day 0 of the estrous cycle) to estimate the progesterone concentration. These samples were centrifuged at 3,000 rpm for 10 min to obtain the serum. These serum samples were then stored at −20°C until further analysis.

### 2.6 Hormonal assay

#### 2.6.1 Serum progesterone assay

The serum progesterone concentration (ng/ml) was estimated on six different days (days −7, 0, 15, 30, 45, and 60) during a feeding trial using an ELISA kit from Cayman Chemicals, USA (582601). The test had a range of 7.8–1,000 pg/ml and a sensitivity of approximately 10 pg/ml (80% B/B0).

#### 2.6.2 Serum estradiol assay

I^125^ diagnostic kits supplied by Immunotech, France and BARC, Mumbai, India, were used to estimate the estrogen concentration in serum samples. The kit's analytical sensitivity for estimating estradiol was < 6 pg/ml. The intra- and inter-assay coefficients of variation for estradiol were 12.1% and 11.2%, respectively.

### 2.7 Hematological parameter

Blood samples were collected from only six ewes fed with flaxseed on days −7, 30, and 60 for convenience. The samples were collected aseptically from the jugular vein into vacutainers. Various hematological parameters such as red blood cells (million/mm^3^), white blood cells (thousand/mm^3^), packed cell volume (PCV) (%), and hemoglobin (g/dl) were estimated according to Benjamin's method (22).

### 2.8 Estimation of blood glucose

Blood samples were collected aseptically on days −7, 0, 15, 30, 45, and 60 from the jugular vein into vacutainers for glucose level estimation using an Accu-Chek Instant Blood Glucose Meter glucometer.

#### 2.8.1 Estimation of serum fatty acid concentration

Free FAs were measured in blood samples on days −7, 0, 15, 30, 45, and 60 of supplementation. Fatty acid methyl esters (FAME) are prepared from lipids in blood samples (23). FAMEs were separated by gas chromatography (GC; 450-GC; Bruker, Billerica, MA, USA) using an SGE Forte GC capillary column (60 m × 0.25 mm × 70 m; BPX70). Helium was used as a carrier gas. Injector and detector temperatures were 260°C and 270°C, respectively. The temperature increase schedule was as follows. After injection, the initial temperature was held at 100°C for 5 min, then, it was increased at a rate of 2°C/min to 240°C and held for 5 min. The sample (1 μl) was injected into the separation solution (with a split ratio of 10:1). Individual FAMEs were identified based on the retention time of 37 fatty acid FAME samples (Supelco, Bellefonte, PA, USA). Peak areas in the chromatogram were calculated and normalized using the response. Individual FA content was expressed as a weight percentage (g/100 g FAME).

### 2.9 Statistical analysis

The data were first checked for adherence to the assumptions of normal distribution using the Shapiro–Wilk test. Standard statistical procedures, according to Snedecor and Cochran (24), were used for the analysis. The analysis was performed using SPSS Statistics (IBM^®^) version 27. Time series data were analyzed using the generalized linear model (GLM) repeated measures analysis of variance (ANOVA), including terms for group, time, and their interactions in the model. Data are shown as mean ± standard error (SE). The statistical significance of the parameters was determined at 95% confidence intervals.

## 3 Results

### 3.1 Reproductive performance

The estrus induction rate was significantly higher (*p* < 0.05) in all the treatment groups than in the control group (Table 2). Group III had a significantly lower (*p* < 0.05) estrus induction interval than the other treatment groups. The control group ewes recorded a significantly lower (*p* < 0.05) conception rate. There is no significant difference (*p* > 0.05) in the gestation length between the control group and the treatment groups. In addition, the control group ewes had a significantly lower lambing rate (*p* < 0.05) than ewes in the treatment groups.

**Table 2:** Effect of linseed supplementation on the reproductive performance of Marwari sheep.

**Groups**	**Total no. of animals (n)**	**Estrus induction rate (%)^$^**	**Estrus induction interval (days)**	**Conception rate (%)^$^**	**Gestation length (days)**	**Lambing rate (%)^$^**	**Twinning %**
I	12	33.33^b^ (4/12)	44.75 ± 1.70^*^	25^b^ (3/12)	148.33 ± 0.88^*^	25^b^ (3/12)	0
II	12	83.33^a^ (10/12)	38.4 ± 0.70^b^	66.67^a^ (8/12)	148.87 ± 0.47^a^	91.66^a^ (11/12)	37.5^a^
III	12	100^a^ (11/12)	35.09 ± 0.28^c^	83.33.90^a^ (10/12)	147.8 ± 0.61^a^	133.33^a^ (16/12)	60^a^
IV	12	66.67^a^ (8/12)	40.37 ± 0.41^a^	66.67^a^ (8/12)	147.75 ± 0.70^a^	91.66^a^ (11/12)	37.5^a^

Means in a column bearing different superscripts (a, b, c) differ significantly at a *p*-value of < 0.05.

^$^Figures in the parenthesis indicate the number of observations.

^*^Not included in the statistical analysis because n was < 6.

### 3.2 Serum progesterone concentration

The mean serum progesterone concentrations (P_4_) for ewes in the control and treatment groups obtained on different days are shown in Table 3. In groups II, III, and IV, the serum progesterone concentration was significantly higher (*p* < 0.05) on treatment day 60 than on days −7, 0, 15, 30, and 45. The serum progesterone concentration increased significantly (*p* < 0.05) in all treatment groups from day 15 onward.

**Table 3:** Effect of linseed supplementation on serum progesterone concentration (ng/ml) of Marwari sheep (mean ± SE).

**Days**	**−7**	**0**	**15**	**30**	**45**	**60**	**Group x Days (*p*-value)**
Group I	0.45^Aa^ ± 0.05	0.32^Aa^ ± 0.02	0.49^Ba^ ± 0.03	1.23^Bb^ ± 0.12	1.41^Bb^ ± 0.44	3.78^Bb^ ± 0.90	0.063
Group II	0.60^Aa^ ± 0.03	0.85^Aa^ ± 0.28	1.16^Aa^ ± 0.03	1.92^Aa^ ± 0.17	3.03^Aa^ ± 0.37	7.05^Ab^ ± 0.94	0.029
Group III	0.57^Aa^ ± 0.01	0.67^Aa^ ± 0.24	1.19^Aa^ ± 0.04	1.47^Aa^ ± 0.19	3.64^Aa^ ± 0.39	8.14^Ab^ ± 0.87	0.004
Group IV	0.28^Aa^ ± 0.02	0.40^Aa^ ± 0.10	1.03^Aa^ ± 0.09	2.07^Aa^ ± 0.15	3.52^Aa^ ± 0.54	7.28^Ab^ ± 0.68	0.002

Means within a row (a, b) and a column (A, B) with different superscript letters differ significantly (*p* < 0.05). Each group had *n* = 12 sheep.

### 3.3 Serum estrogen concentration

The mean serum estrogen concentrations (E_2_) for ewes in the control and treatment groups obtained on different days are shown in Table 4. There was no significant difference in the mean serum estrogen concentration in group I between sampling days (*p* > 0.05). On treatment days 15 and 30, the serum estrogen concentration was significantly higher (*p* < 0.05) in all the treatment groups than in the control group.

**Table 4:** Effect of linseed supplementation on the serum estrogen concentration (pg/ml) of Marwari sheep (mean ± SE).

**Days**	**−7**	**0**	**15**	**30**	**45**	**60**	**Group x Days (*p-value*)**
Group I	3.36^Aa^ ± 0.15	3.08^Aa^ ± 0.42	3.69^Ba^ ± 0.77	3.91^Ba^ ± 0.22	4.11^Aa^ ± 0.66	2.38^Aa^ ± 0.22	0.405
Group II	3.55^Aa^ ± 0.15	3.59^Aa^ ± 0.36	6.39^Ab^ ± 0.90	7.15^Ab^ ± 0.44	6.20^Ab^ ± 1.31	3.15^Aa^ ± 0.62	0.552
Group III	3.38^Aa^ ± 0.19	4.02^Aa^ ± 0.29	7.26^Ab^ ± 1.04	8.78^Ab^ ± 1.14	6.83^Ab^ ± 1.05	3.00^Aa^ ± 0.69	0.116
Group IV	3.31^Aa^ ± 0.39	3.72^Aab^ ± 0.37	7.16^Ac^ ± 0.76	7.52^Ac^ ± 1.21	6.06^Abc^ ± 1.21	2.79^Aa^ ± 0.56	0.429

Means within a row (a, b, c) and a column (A, B) with different superscript letters differ significantly (*p* < 0.05). Each group had *n* = 12 sheep.

### 3.4 Hemoglobin levels

The mean serum hemoglobin concentrations for ewes in the control and treatment groups obtained on different days are presented in Table 5. The hemoglobin concentrations did not differ significantly (*p* > 0.05) in the control group and group IV on different sampling days. The hemoglobin level was significantly higher (*p* < 0.05) in all the treatment groups than in the control group on day 60.

**Table 5:** Effect of linseed supplementation on the hemoglobin levels (g/dl) of Marwari sheep (mean ± SE).

**Days**	**−7**	**30**	**60**	**Group x Days (*p-*value)**
Group-I	11.24^Aa^ ± 0.44	11.52^Aa^ ± 0.41	11.92^Ba^ ± 0.37	0.928
Group-II	11.30^Ab^ ± 0.55	12.00^Aab^ ± 0.60	13.6^Aa^ ± 0.46	0.017
Group-III	11.23^Ab^ ± 0.30	12.50^Ab^ ± 0.36	14.33^Aa^ ± 0.46	0.001
Group-IV	11.37^Aa^ ± 0.30	12.31^Aa^ ± 0.16	13.83^Aa^ ± 0.33	0.042

Means within a row (a, b) and a column (A, B) with different superscript letters differ significantly (*p* < 0.05). Each group had *n* = 12 sheep.

### 3.5 Packed cell volume

The mean concentrations of serum PCV for ewes in the control and treatment groups obtained on different days are presented in Table 6. The PCV did not differ significantly (*p* > 0.05) in the control group on any sampling days, whereas on days 30 and 60, the PCVs in all the treatment groups were significantly higher (*p* < 0.05) than in the control group.

**Table 6:** Effect of linseed supplementation on packed cell volume (%) of Marwari sheep (mean ± SE).

**Days**	**−7**	**30**	**60**	**Group x Days (*p-*value)**
Group-I	31.18^Aa^ ± 0.58	31.08^Ba^ ± 0.42	31.50^Ba^ ± 0.39	0.488
Group-II	31.47^Ab^ ± 0.59	33.97^Aa^ ± 0.56	35.58^Aa^ ± 0.79	0.005
Group-III	31.45^Ab^ ± 0.48	36.01^Aa^ ± 0.84	37.02^Aa^ ± 0.52	0.233
Group-IV	31.15^Ac^ ± 0.37	33.88^Ab^ ± 0.47	35.78^Aa^ ± 0.54	0.353

Means within a row (a, b, c) and a column (A, B) with different superscript letters differ significantly (*p* < 0.05). Each group had *n* = *n* = 12 sheep.

### 3.6 Total leukocyte count

The mean concentrations of TLC for ewes in the control and treatment groups obtained on different days are presented in Table 7. The TLC did not differ significantly (*p* > 0.05) in the control group on any sampling days. The TLC was significantly higher (*p* < 0.05) in groups III and IV on day 30 than in the control group and group II.

**Table 7:** Effect of linseed supplementation on the serum total leukocyte count (thousand/mm^3^) of Marwari sheep (mean ± SE).

**Days**	**−7**	**30**	**60**	**Group x Days (*p-*value)**
Group-I	8.17^Aa^ ± 0.20	8.32^Ba^ ± 0.28	8.67^Ba^ ± 0.30	0.993
Group-II	8.24^Ab^ ± 0.19	8.55^Bb^ ± 0.32	9.98^Aa^ ± 0.56	0.150
Group-III	8.48^Ab^ ± 0.35	9.42^Aab^ ± 0.46	9.65^ABa^ ± 0.35	0.001
Group-IV	8.75^Ab^ ± 0.44	9.47^Ab^ ± 0.24	10.87^Aa^ ± 0.39	0.035

Means within a row (a, b) and a column (A, B) with different superscript letters differ significantly (*p* < 0.05). Each group had *n* = 12 sheep.

### 3.7 Total erythrocyte count

The mean concentrations of TEC for ewes in the control and treatment groups obtained on different days are presented in Table 8. There was no significant difference (*p* < 0.05) in the TEC in groups I and II on different sampling days. On days −7 and 30, there was no significant difference (*p* > 0.05) among the groups. However, on day 60, the TEC was significantly higher (*p* < 0.05) in group III than in the control group.

**Table 8:** Effect of linseed supplementation on the serum total erythrocyte count (million/mm^3^) of Marwari sheep (mean ± SE).

**Days**	**−7**	**30**	**60**	**Group x Days (*p-*value)**
Group-I	10.47^Aa^ ± 0.57	10.97^Aa^ ± 0.62	11.23^Ba^ ± 0.72	0.086
Group-II	10.40^Aa^ ± 0.71	11.28^Aa^ ± 0.50	12.15^ABa^ ± 0.50	0.366
Group-III	10.35^Ac^ ± 0.59	12.03^Ab^ ± 0.29	13.50^Aa^ ± 0.23	0.206
Group-IV	10.45^Ab^ ± 0.61	11.45^Aab^ ± 0.54	12.80^ABa^ ± 0.63	0.250

Means within a row (a, b, c) and a column (A, B) with different superscript letters differ significantly (*p* < 0.05). Each group had *n* = 12 sheep.

### 3.8 Glucose

Serum glucose concentrations (mg/dl) for ewes in the control and treatment groups obtained on different days are presented in Table 9. There was no significant difference (*p* ≥ 0.05) in the glucose concentrations among the groups on days −7, 0, 15, and 30. However, on days 45 and 60, the serum glucose concentrations were significantly lower (*p* < 0.05) in group I than in groups II, III, and IV.

**Table 9:** Effect of linseed supplementation on the serum glucose concentration (mg/dl) of Marwari sheep (mean ± SE).

**Days**	**−7**	**0**	**15**	**30**	**45**	**60**	**Group x Days (*p-*value)**
Group-I	55.83^Aa^ ± 3.68	56.50^Aa^ ± 3.87	57.17^Aa^ ± 3.77	58.00^Aa^ ± 3.86	58.67^Ba^ ± 3.86	59.17^Ba^ ± 3.88	0.536
Group-II	56.00^Ab^ ± 3.54	56.67^Ab^ ± 3.56	62.50^Aab^ ± 2.31	64.50^Aab^ ± 2.61	67.17^Aa^ ± 2.17	69.67^Aa^ ± 2.01	0.320
Group-III	56.50^Ac^ ± 3.31	57.33^Ac^ ± 3.40	63.67^Aab^ ± 2.47	65.83^Aab^ ± 3.09	69.17^Aab^ ± 1.99	73.50^Aa^ ± 1.96	0.144
Group-IV	56.83^Ac^ ± 3.68	57.33^Ac^ ± 3.58	59.50^Aab^ ± 3.77	64.67^Aab^ ± 3.01	68.17^Aab^ ± 2.15	71.17^Aa^ ± 1.96	0.962

Means within a row (a, b, c) and a column (A, B) with different superscript letters differ significantly (*p* < 0.05). Each group had *n* = 12 sheep.

### 3.9 Total MUFA

The mean serum MUFA concentrations (g/100 g total FA) for ewes in the control and treatment groups obtained on different days are presented in Table 10. The serum MUFA concentrations did not differ significantly (*p* > 0.05) in the control group on any sampling days, whereas it decreased significantly (*p* < 0.05) in all the treatment groups from day 15 onward.

**Table 10:** Effect of linseed supplementation on total MUFA concentrations (g/100 g of total fatty acids) of Marwari sheep (mean ± SE).

**Days**	**−7**	**0**	**15**	**30**	**45**	**60**	**Group x Days (*p-*value)**
Group-I	27.16^Aa^ ± 0.40	26.33^Aa^ ± 0.32	26.15^Aa^ ± 0.37	25.82^Aa^ ± 0.30	25.37^Aa^ ± 0.31	25.09^Aa^ ± 0.28	0.063
Group-II	27.02^Aa^ ± 0.32	27.18^Aa^ ± 0.48	23.51^Bb^ ± 0.34	23.54^Bb^ ± 0.61	22.85^Bb^ ± 0.41	22.06^Cb^ ± 0.25	0.001
Group-III	27.69^Aa^ ± 0.31	27.99^Aa^ ± 0.55	23.58^Bb^ ± 0.45	22.48^Bb^ ± 0.30	22.90^Bb^ ± 0.29	23.38^Bb^ ± 0.27	0.001
Group-IV	27.09^Aa^ ± 0.38	26.71^Aa^ ± 0.26	23.61^Bb^ ± 0.28	23.50^Bb^ ± 0.36	23.85^Bb^ ± 0.43	23.44^Bb^ ± 0.28	0.001

Means within a row (a, b) and a column (A, B, C) with different superscript letters differ significantly (*p* < 0.05). Each group had *n* = 12 sheep.

### 3.10 Total PUFA

The mean serum PUFA concentrations (g/100 g of total FA) for ewes in the control and treatment groups obtained on different days are presented in Table 11. The mean serum PUFA concentration in the control group did not differ significantly (*p* < 0.05) throughout the study, whereas it increased significantly (*p* < 0.05) in all treatment groups from day 15 onward.

**Table 11:** Effect of linseed supplementation on total PUFA concentrations (g/100 g total fatty acids) of Marwari sheep (mean ± SE).

**Days**	**−7**	**0**	**15**	**30**	**45**	**60**	**Group x Days (*p*-value)**
Group-I	26.67^Aa^ ± 0.59	27.01^Aa^ ± 0.41	27.01^Ba^ ± 0.41	27.09^Ba^ ± 0.56	27.38^Ba^ ± 0.44	27.72^Ba^ ± 0.24	0.840
Group-II	27.44^Aa^ ± 0.44	27.65^Aa^ ± 0.36	30.44^Ab^ ± 0.29	33.29^Ac^ ± 0.47	35.85^Ad^ ± 0.73	38.45^Ae^ ± 0.30	0.001
Group-III	26.95^Aa^ ± 0.66	26.93^Aa^ ± 0.48	30.45^Ab^ ± 0.40	32.58^Ac^ ± 0.48	35.11^Ad^ ± 0.60	37.40^Ae^ ± 0.80	0.001
Group-IV	27.75^Aa^ ± 0.37	27.66^Aa^ ± 0.25	30.80^Ab^ ± 0.32	32.9^Ac^ ± 0.36	36.37^Ad^ ± 0.50	38.07^Ae^ ± 0.72	0.001

Means within a row (a, b, c, d, e) and a column (A, B) with different superscript letters differ significantly (*p* < 0.05). Each group had *n* = 12 sheep.

## 4 Discussion

Previous studies that investigated the effects of ω-3 PUFA on the reproductive performance of pigs, cows, buffalos, sheep, and goats reported contradictory results. These inconsistencies in the results are primarily caused by differences in the animal species, the source and dosage of ω-3 FA, and the duration of feeding. Despite the presence of abundant literature, there is little evidence that linseed supplementation affects sheep reproduction in tropical and subtropical climates. Thus, this study aimed to evaluate the effects of dietary supplementation of ω-3 PUFA derived from linseed on the reproductive parameters, critical hormonal profiles, and biochemistry in sheep during the breeding season in subtropical climates.

In the present study, the estrus induction rate was significantly higher in all treatment groups than in the control group. Similarly, the estrus induction interval was significantly shorter in group III. The control ewes recorded significantly lower conception rates, and the lambing rates were significantly higher in linseed-supplemented sheep. Mahla et al. (2) demonstrated that supplementation of fish oil with n-3 PUFAs for ewes improved their follicle numbers, ovulation rates, and twinning percentages. El-Shahata and Abo-Elmaaty (25) reported increased ovarian preovulatory follicles and ovulation rates in ewes after supplementation with calcium salts of long-chain FAs. According to Mahla et al. (26), supplementing ω-3 PUFA-rich fish oil in goats significantly increased their follicle count, ovulation rate, and kidding rate as well as the follicle size. In a study conducted by Garcia-Bojalil et al. (27), supplementation of calcium salts of long-chain FAs increased the number of CLs, decreased the time for the initial increase in progesterone, and restored the pattern of accumulated plasma progesterone concentrations. Moreover, the use of FA calcium soaps during flushing has been confirmed to be effective in improving fertility and lambing rate (28). Similarly, n-3 PUFAs improved the conception rate, follicular turnover, and growth in cattle (11, 12, 29). Some studies also reported that supplementation with n-3 PUFA increased the number of small (30, 31), medium (29, 32), and large (33) follicles in cattle, indicating that n-3 PUFA affects the follicular dynamics. Cattle supplemented with flaxseed oil and fish oil were found to have increased number of small follicles (34, 35). According to Ambrose et al. (11), cows fed with flaxseed oil showed a greater conception rate in a fixed timed artificial insemination (AI) than cows fed with sunflower oil. Similarly, Dirandeh et al. (36) observed a 66.7% increase in the conception rate in cows supplemented with flaxseed compared to that in control cows. Nazir et al. (16) observed increased conception rates in buffaloes supplemented with flaxseed on day 63 post-AI than in the control buffaloes. Akbarinejad et al. (37) reported no change in their findings, which is in contrast to the findings of the present study. This difference in results could be attributed to multiple factors, including the duration of PUFA supplementation, the source of PUFA, climate variation, and breed differences.

PUFAs are precursors of PGs, which play multiple roles in animal reproduction (9). Mammals must obtain PUFAs from their diet because they lack the enzymes essential to synthesize PUFAs internally (38). Arachidonic acid (AA), an n-6 PUFA, serves as the precursor for pro-inflammatory 2-series PGs. Conversely, animals can also experience anti-inflammatory effects by consuming food rich in n-3 PUFAs such as eicosapentaenoic acid (20:5 n-3) and docosahexaenoic acid (22:6 n-3). According to Abayasekara and Wathes (39), PG metabolism can be affected by the ratio of n-6 to n-3 PUFAs in the diet. *In vivo* and *in vitro* experiments have established that n-3 PUFAs reduce endometrial PG synthesis, thereby contributing to embryo survival (40–42). PUFAs are involved in a number of processes that play crucial roles in the normal fluidity of cell membranes and intracellular signaling (9), as well as in ovulation, fertilization, and parturition (39).

The present study demonstrated that ω-3 PUFA-rich linseed supplementation did not affect gestation length in sheep. This finding is consistent with studies in sows supplemented with ω-3 PUFA-rich flaxseed around the breeding time, which did not alter the pregnancy length (20, 43). Mattos et al. (44) revealed that supplementing cows with fish oil during the peri-partum period had no effect on the gestation length. By contrast, the gestation length was extended by 2 days (147.5 vs. 145.5 days) in ewes supplemented with fish oil in the late gestation period compared to those supplemented with Megalac (14). These findings may be explained by the fact that, in the present study, diets rich in ω-3 PUFA were supplemented during the early gestation period, whereas when ω-3 PUFA was supplemented during the late gestation period, prolonged gestation has been observed. A diet supplemented with PUFAs can affect the gestation length by altering the types and quantities of PGs synthesized, which are essential for parturition (45).

The present study demonstrated that the serum estrogen and progesterone concentrations were significantly affected by linseed supplementation. These results are in accordance with previous studies conducted on PUFA-fed sheep (25, 46) and goats (47). Sklan et al. (48) showed that cows fed with calcium salts of FAs produced higher progesterone during the estrous cycle than control cows. According to Petit and Twagiramungu (12) and Dirandeh et al. (36), cows that received flaxseed supplements had higher P_4_ levels. An increased number of CLs and improved ovarian steroidogenesis may account for this increase in progesterone levels. According to Hawkins et al. (49), increased P_4_ in cows fed with calcium salts of long-chain FAs may be related to reduced P_4_ clearance from the plasma rather than increased luteal cell uptake of cholesterol. Nevertheless, Robinson et al. (50) observed lower plasma progesterone in cows treated with PUFAs in the early luteal phase. Higher proliferator-activated receptors have been linked to higher plasma concentrations of eicosapentaenoic acid (51), which could lead to a decrease in P_4_ clearance (52). Conception rates may also increase with PUFA feeding because of the increased production P_4_ due to increased CL and decreased embryo mortality (8, 12, 53, 54). FA supplementation in cyclic ewes increased the levels of progesterone in the blood and the size and number of ovarian follicles (5). According to Rawlings et al. (55) and Bartlewski et al. (56, 57), feeding an FA-concentrated diet increased the mean serum estrogen (pg/ml) content in sheep by three to four times. The unsaturated FA content of linseed has been shown to improve the physical properties of follicles and oocytes (58, 59).

In the present study, the hemoglobin concentrations in the treatment groups increased significantly on day 60 compared to that in the control group; however, no effect was recorded on day 30. Unfortunately, studies on sheep are not available in the literature that could be used for comparison. However, the pattern of change in the hemoglobin level was similar to that of the observations of other studies that used flaxseed oil in rats (60, 61). The addition of linseed oil to broiler chicks produced a non-significant shift in the concentrations of hemoglobin (62). In terms of PCV, the results of the present study showed that the percentage of PCV increased on days 30 and 60 of linseed supplementation. Improvement of PCV due to linseed supplementation could be due to its high content of PUFA, which protects the cell membrane of red blood corpuscles. In another study on rats, flaxseed oil improved the TEC and PCV owing to its high content of n-3 PUFA (63). Similarly, in the case of the TLC and TEC, a significant effect of linseed supplementation was observed on day 60. Similar profiles of the TEC were recorded in other studies on linseed-fed lambs (64–66). Furthermore, a study on Iraqi Awassi lambs fed with flaxseed oil showed improved hemoglobin concentration, red blood cell count, and blood glucose levels as well as decreased TLC after oral dosing (67). Supplementing with linseed may result in reticulocytosis, induce polycythemia, and boost erythropoietic activity in the liver and spleen. Shunthwal et al. (68) showed that feeding broiler chicks linseed oil enhanced their blood parameters and, consequently, the n6/n3 ratio of FAs in the body system.

The findings of the present study indicated a statistically significant increase in blood glucose levels in the treatment groups on days 45 and 60 compared to the control group. These findings are consistent with the results of Kia and Safdar (46). However, fish oil did not significantly affect the blood glucose levels in sheep, according to Mahla et al. (2). FAs can have a direct impact on insulin secretion triggered by glucose. Saturated FAs are insulinogenic, implying that they have a stronger effect on insulin secretion than unsaturated FAs (69, 70). Furthermore, lipid supplements with various FA compositions can alter the release of gastrointestinal peptides such as glucagon-like peptide 1 and glucose-dependent insulinotropic peptide 1. Thus, as observed in Holstein cows, this alteration may improve glucose-mediated insulin secretion (71). Cows fed diets enriched with flaxseed (linseed) and fish oil had higher plasma glucose levels (29, 72). Conversely, ewes fed diets enriched with flaxseed oil did not show a significant change in glucose levels (73).

In the present study, linseed supplementation significantly reduced serum MUFA concentration but significantly increased serum PUFA concentration from day 15 of supplementation. There is a lack of literature on the effect of feeding linseed to sheep on their serum MUFA and PUFA profiles. Mahla et al. (2) found that feeding PUFA to ewes significantly affected their plasma total cholesterol as well as high- and low-density lipoprotein-cholesterol concentrations. Similarly, Abuelfatah et al. (74) found that feeding whole linseed to goats increased the concentration of PUFA in the rumen. According to Singh et al. (75, 76), feeding boars linseed oil can boost antioxidant indicators and significantly reduce lipid peroxidation in their seminal plasma and serum. Furthermore, because linseed oil contains a higher concentration of these FAs than other oils, feeding sheep linseed oil may alter their serum PUFA and MFA profiles. This finding is consistent with the finding of Petit et al. (29). Although the present study provides the first comprehensive results on the reproductive performance of sheep fed linseed in subtropical climates, it has limitations related to a small number of animals and a short duration of study. It would be interesting to investigate the long-term effects of flaxseed supplementation. Furthermore, future studies focusing on follicular dynamics, follicular atresia, and embryonic development would be interesting.

## 5 Conclusion

Supplementing linseed to nulliparous ewes for 2 months during the breeding season in subtropical climates improved their reproductive efficiency by modulating the hormonal and biochemical milieu. Ewes that were fed linseed (15%) had a significantly higher rate of estrus induction, a shorter estrus induction interval, and an increased rate of conception and twinning. Simultaneously, linseed supplementation resulted in increased serum P_4_ concentration, decreased serum MUFA levels, and increased serum PUFA levels. The study suggests that strategic dietary adjustments, such as the inclusion of 15% linseed during the breeding season, can significantly enhance reproductive outcomes in sheep farmed in subtropical climates.

## Data availability statement

The original contributions presented in the study are included in the article/supplementary material, further inquiries can be directed to the corresponding authors.

## Ethics statement

The animal study was approved by Institute Animal Ethics Committee, NDVSU-College of Veterinary Science and Animal Husbandry. The study was conducted in accordance with the local legislation and institutional requirements.

## Author contributions

PA: Writing – original draft, Conceptualization, Data curation, Investigation, Validation. JR: Conceptualization, Writing – original draft, Project administration, Resources, Supervision. AS: Conceptualization, Writing – original draft, Data curation, Investigation. BO: Investigation, Writing – original draft, Methodology, Supervision, Validation. AJ: Investigation, Methodology, Writing – original draft, Formal analysis. AB: Investigation, Writing – original draft. NB: Investigation, Writing – original draft, Conceptualization, Methodology. MA: Conceptualization, Writing – original draft. AR: Writing – original draft, Methodology. AS: Writing – original draft, Data curation, Investigation. SP: Writing – original draft, Formal analysis, Writing – review & editing. AM: Writing – review & editing. RK: Writing – review & editing. JC: Writing – review & editing. MS: Writing – review & editing.
